# Supplementary material for: Late-life fitness gains and reproductive death in Cardiocondyla obscurior ants
Source: eLife. 2022 Apr 6;11:e74695. doi: 10.7554/eLife.74695 (PMC8986319; doi:10.7554/eLife.74695)
Supplement: Supplementary file 1. — (A) Estimates calculated for 10, 20, and 30 workers using a glmmTMB model with Gaussian distribution and ‘setup date,’ ‘experimental box,’ ‘box,’ and ‘nest of origin’ as random factors for (Queen*c)/[(Queen*c) + Worker]. The coefficient c as the average dry weight of queens over workers to the power conversion factor of 0.6–1 (see text). All comparisons between 10 and 20 workers treatments are statistically significant (p<0.001), but not between 20 and 30 workers. (B) The 15 most significant enriched Gene Ontology (GO) terms per type in differentially expressed genes (DEGs) enriched in middle-aged queens in the head-thorax tissue. BP: Biological Processes; CC: cellular component; MF: molecular functions. (C) The 15 most significant enriched GO terms per type in DEGs enriched in prope mortem queens in the head-thorax tissue. (D) The 15 most significant enriched GO terms per type in DEGs enriched in prope mortem queens in the gaster tissue. (E) The 15 most significant enriched GO terms per type in DEGs enriched in middle-aged queens in the gaster tissue. (F) Shared enriched GO terms for both tissues (head-thorax and gaster) enriched in prope mortem queens. (G) Shared enriched GO terms for both tissues (head-thorax and gaster) enriched in middle-aged queens. (H) Deviance information criterion (DIC) of three tested models (logistic, Gompertz, and Weibull) for the age-specific mortality of ant queens. Using the function multibasta of the R package BaSTA (Survival Bayesian Trajectory Analysis, v. 1.9.5). The model with the lowest DIC value is assumed to provide the best fit. (I) Estimated coefficients of age-specific mortality of ant queens. Using a logistic model and the R package BaSTA (Survival Bayesian Trajectory Analysis, v. 1.9.5). [file elife-74695-supp1.docx]

Late-life fitness gains and reproductive death in *Cardiocondyla obscurior* ants

Authors: Jaimes-Nino, LM^1^, Heinze, J^1^; Oettler, J^1*^

Affiliation: ^1^Zoologie/Evolutionsbiologie, Universität Regensburg, 93053 Germany

^*^Correspondence: Luisa M. Jaimes-Nino (jaimes.luisa@outlook.com) and Jan Oettler (joettler@gmail.com).

^2^Lead Contact: Further information and requests for resources should be directed to and will be fulfilled by the Lead Contact, Luisa M. Jaimes Nino (jaimes.luisa@outlook.com).

Keywords: aging, selection shadow, senescence, social insects

^3^Twitter: @luisamjaimesn

This PDF file includes:

Suppementary files 1 A-I

**Supplementary File 1A. Estimates calculated for 10, 20 and 30 workers using a glmmTMB model with Gaussian distribution and ‘setup date’, ‘experimental box’, ‘box’ and ‘nest of origin’ as random factors for (Queen*c) / [(Queen*c) + Worker].** The coefficient c as the dry average weight measurements of queen over workers to the power conversion factor of 0.6-1 (see text). All comparisons between 10 and 20 workers treatments are statistically significant (p<0.001), but not between 20 and 30 workers.

| Power conversion | Estimates | | |
| --- | --- | --- | --- |
|  | 10 workers | 20 workers | 30 workers |
| 0.6 | 0.091 | 0.092 | 0.083 |
| 0.7 | 0.095 | 0.095 | 0.086 |
| 0.8 | 0.099 | 0.098 | 0.088 |
| 0.9 | 0.103 | 0.101 | 0.091 |
| 1 | 0.107 | 0.104 | 0.094 |

**Supplementary File 1B.** **The 15 most significant enriched GO-terms per type in DEGs enriched in middle-aged queens in the head-thorax tissue.** BP = Biological processes, CC = Cellular component, and MF = Molecular functions.

| Type | GO.ID | Term | Annotated | Significant | Expected | Fisher |
| --- | --- | --- | --- | --- | --- | --- |
| BF | GO:0006412 | translation | 200 | 78 | 21,65 | < 1e-30 |
| BF | GO:0006099 | tricarboxylic acid cycle | 17 | 6 | 1,84 | 0,0001 |
| BF | GO:0006816 | calcium ion transport | 15 | 7 | 1,62 | 0,00011 |
| BF | GO:0006614 | SRP-dependent cotranslational protein ta... | 8 | 4 | 0,87 | 0,00034 |
| BF | GO:1902600 | proton transmembrane transport | 26 | 12 | 2,81 | 0,00036 |
| BF | GO:0006414 | translational elongation | 14 | 5 | 1,52 | 0,00058 |
| BF | GO:0006357 | regulation of transcription by RNA polym... | 28 | 8 | 3,03 | 0,00065 |
| BF | GO:0030150 | protein import into mitochondrial matrix | 5 | 3 | 0,54 | 0,00108 |
| BF | GO:0006511 | ubiquitin-dependent protein catabolic pr... | 50 | 10 | 5,41 | 0,00113 |
| BF | GO:0007186 | G protein-coupled receptor signaling pat... | 115 | 15 | 12,45 | 0,00128 |
| BF | GO:0006470 | protein dephosphorylation | 35 | 7 | 3,79 | 0,00131 |
| BF | GO:0007154 | cell communication | 396 | 49 | 42,87 | 0,00211 |
| BF | GO:0042773 | ATP synthesis coupled electron transport | 10 | 5 | 1,08 | 0,00236 |
| BF | GO:0031032 | actomyosin structure organization | 2 | 2 | 0,22 | 0,00239 |
| BF | GO:0008615 | pyridoxine biosynthetic process | 2 | 2 | 0,22 | 0,00239 |
| CC | GO:0005840 | ribosome | 126 | 67 | 14,23 | < 1e-30 |
| CC | GO:0019773 | proteasome core complex, alpha-subunit c... | 7 | 4 | 0,79 | 0,00025 |
| CC | GO:0005737 | cytoplasm | 378 | 83 | 42,69 | 0,00025 |
| CC | GO:0015934 | large ribosomal subunit | 13 | 7 | 1,47 | 0,00046 |
| CC | GO:0005874 | microtubule | 9 | 4 | 1,02 | 0,00082 |
| CC | GO:0000276 | mitochondrial proton-transporting ATP sy... | 5 | 3 | 0,56 | 0,00141 |
| CC | GO:0042025 | host cell nucleus | 73 | 11 | 8,24 | 0,00158 |
| CC | GO:0005839 | proteasome core complex | 14 | 8 | 1,58 | 0,00259 |
| CC | GO:0062023 | collagen-containing extracellular matrix | 2 | 2 | 0,23 | 0,00286 |
| CC | GO:0046658 | anchored component of plasma membrane | 2 | 2 | 0,23 | 0,00286 |
| CC | GO:0016593 | Cdc73/Paf1 complex | 2 | 2 | 0,23 | 0,00286 |
| CC | GO:0000814 | ESCRT II complex | 2 | 2 | 0,23 | 0,00286 |
| CC | GO:0016020 | membrane | 1234 | 142 | 139,36 | 0,00414 |
| CC | GO:0005739 | mitochondrion | 87 | 28 | 9,83 | 0,00725 |
| CC | GO:0005787 | signal peptidase complex | 3 | 2 | 0,34 | 0,00828 |
| MF | GO:0003735 | structural constituent of ribosome | 126 | 68 | 14,68 | < 1e-30 |
| MF | GO:0005515 | protein binding | 1576 | 206 | 183,57 | < 1e-30 |
| MF | GO:0005509 | calcium ion binding | 146 | 19 | 17,01 | 0,00012 |
| MF | GO:0004784 | superoxide dismutase activity | 3 | 3 | 0,35 | 0,00012 |
| MF | GO:0005524 | ATP binding | 593 | 50 | 69,07 | 0,00019 |
| MF | GO:0003924 | GTPase activity | 104 | 15 | 12,11 | 0,00019 |
| MF | GO:0003676 | nucleic acid binding | 955 | 129 | 111,23 | 0,00024 |
| MF | GO:0005200 | structural constituent of cytoskeleton | 8 | 4 | 0,93 | 0,00036 |
| MF | GO:0004672 | protein kinase activity | 216 | 21 | 25,16 | 0,00067 |
| MF | GO:0046982 | protein heterodimerization activity | 33 | 7 | 3,84 | 0,00101 |
| MF | GO:0042302 | structural constituent of cuticle | 43 | 8 | 5,01 | 0,00112 |
| MF | GO:0003824 | catalytic activity | 2228 | 279 | 259,51 | 0,00132 |
| MF | GO:0009055 | electron transfer activity | 43 | 18 | 5,01 | 0,00141 |
| MF | GO:0016757 | glycosyltransferase activity | 84 | 15 | 9,78 | 0,00145 |
| MF | GO:0051539 | 4 iron, 4 sulfur cluster binding | 11 | 4 | 1,28 | 0,00152 |

**Supplementary File 1C.** **The 15 most significant enriched GO-terms per type in DEGs enriched in *prope mortem* queens in the head-thorax tissue.** BP = Biological processes, CC = Cellular component., and MF = Molecular functions.

| Type | GO.ID | Term | Annotated | Significant | Expected | Fisher |
| --- | --- | --- | --- | --- | --- | --- |
| BF | GO:0003735 | structural constituent of ribosome | 126 | 68 | 14,68 | < 1e-30 |
| BF | GO:0005515 | protein binding | 1576 | 206 | 183,57 | < 1e-30 |
| BF | GO:0005509 | calcium ion binding | 146 | 19 | 17,01 | 0,00012 |
| BF | GO:0004784 | superoxide dismutase activity | 3 | 3 | 0,35 | 0,00012 |
| BF | GO:0005524 | ATP binding | 593 | 50 | 69,07 | 0,00019 |
| BF | GO:0003924 | GTPase activity | 104 | 15 | 12,11 | 0,00019 |
| BF | GO:0003676 | nucleic acid binding | 955 | 129 | 111,23 | 0,00024 |
| BF | GO:0005200 | structural constituent of cytoskeleton | 8 | 4 | 0,93 | 0,00036 |
| BF | GO:0004672 | protein kinase activity | 216 | 21 | 25,16 | 0,00067 |
| BF | GO:0046982 | protein heterodimerization activity | 33 | 7 | 3,84 | 0,00101 |
| BF | GO:0042302 | structural constituent of cuticle | 43 | 8 | 5,01 | 0,00112 |
| BF | GO:0003824 | catalytic activity | 2228 | 279 | 259,51 | 0,00132 |
| BF | GO:0009055 | electron transfer activity | 43 | 18 | 5,01 | 0,00141 |
| BF | GO:0016757 | glycosyltransferase activity | 84 | 15 | 9,78 | 0,00145 |
| BF | GO:0051539 | 4 iron, 4 sulfur cluster binding | 11 | 4 | 1,28 | 0,00152 |
| CC | GO:0030117 | membrane coat | 21 | 5 | 2,69 | 0,00051 |
| CC | GO:0005737 | cytoplasm | 378 | 43 | 48,34 | 0,00057 |
| CC | GO:0005643 | nuclear pore | 6 | 3 | 0,77 | 0,00102 |
| CC | GO:0000124 | SAGA complex | 2 | 2 | 0,26 | 0,00146 |
| CC | GO:0008290 | F-actin capping protein complex | 2 | 2 | 0,26 | 0,00146 |
| CC | GO:0032040 | small-subunit processome | 7 | 3 | 0,9 | 0,00173 |
| CC | GO:0005615 | extracellular space | 9 | 3 | 1,15 | 0,00392 |
| CC | GO:0016459 | myosin complex | 18 | 4 | 2,3 | 0,0042 |
| CC | GO:0005685 | U1 snRNP | 3 | 2 | 0,38 | 0,00427 |
| CC | GO:0000813 | ESCRT I complex | 3 | 2 | 0,38 | 0,00427 |
| CC | GO:0016592 | mediator complex | 21 | 4 | 2,69 | 0,0075 |
| CC | GO:0005885 | Arp2/3 protein complex | 5 | 2 | 0,64 | 0,01352 |
| CC | GO:0005667 | transcription regulator complex | 30 | 7 | 3,84 | 0,01724 |
| CC | GO:0005815 | microtubule organizing center | 6 | 2 | 0,77 | 0,01978 |
| CC | GO:0005680 | anaphase-promoting complex | 7 | 2 | 0,9 | 0,02699 |
| MF | GO:0005515 | protein binding | 1576 | 216 | 195,1 | < 1e-30 |
| MF | GO:0004842 | ubiquitin-protein transferase activity | 38 | 8 | 4,7 | 0,00011 |
| MF | GO:0003682 | chromatin binding | 8 | 4 | 0,99 | 0,00016 |
| MF | GO:0003824 | catalytic activity | 2228 | 222 | 275,81 | 0,00021 |
| MF | GO:0035091 | phosphatidylinositol binding | 27 | 6 | 3,34 | 0,0006 |
| MF | GO:0004675 | transmembrane receptor protein serine/th... | 5 | 3 | 0,62 | 0,00061 |
| MF | GO:0003700 | DNA-binding transcription factor activit... | 119 | 13 | 14,73 | 0,001 |
| MF | GO:0008017 | microtubule binding | 30 | 6 | 3,71 | 0,00108 |
| MF | GO:0051015 | actin filament binding | 13 | 4 | 1,61 | 0,00139 |
| MF | GO:0004715 | non-membrane spanning protein tyrosine k... | 2 | 2 | 0,25 | 0,00162 |
| MF | GO:0004832 | valine-tRNA ligase activity | 2 | 2 | 0,25 | 0,00162 |
| MF | GO:0000049 | tRNA binding | 8 | 3 | 0,99 | 0,00313 |
| MF | GO:0004714 | transmembrane receptor protein tyrosine ... | 9 | 3 | 1,11 | 0,00455 |
| MF | GO:0018024 | histone-lysine N-methyltransferase activ... | 9 | 3 | 1,11 | 0,00455 |
| MF | GO:0004000 | adenosine deaminase activity | 3 | 2 | 0,37 | 0,00473 |

**Supplementary File 1D**. The 15 most significant enriched GO-terms per type in DEGs enriched *in prope mortem* queens in the gaster tissue. BP = Biological processes, CC = Cellular component., and MF = Molecular functions.

| Type | GO.ID | Term | Annotated | Significant | Expected | Fisher |
| --- | --- | --- | --- | --- | --- | --- |
| BF | GO:0043248 | proteasome assembly | 6 | 4 | 1,14 | 0,00028 |
| BF | GO:0042147 | retrograde transport, endosome to Golgi | 3 | 3 | 0,57 | 0,00031 |
| BF | GO:0007186 | G protein-coupled receptor signaling pat... | 115 | 19 | 21,86 | 0,00039 |
| BF | GO:0006367 | transcription initiation from RNA polyme... | 12 | 5 | 2,28 | 0,00075 |
| BF | GO:0007264 | small GTPase mediated signal transductio... | 36 | 8 | 6,84 | 0,00086 |
| BF | GO:0007018 | microtubule-based movement | 39 | 9 | 7,41 | 0,00096 |
| BF | GO:0006869 | lipid transport | 37 | 12 | 7,03 | 0,00115 |
| BF | GO:0015914 | phospholipid transport | 10 | 5 | 1,9 | 0,00117 |
| BF | GO:0006904 | vesicle docking involved in exocytosis | 8 | 4 | 1,52 | 0,00117 |
| BF | GO:0000077 | DNA damage checkpoint signaling | 4 | 3 | 0,76 | 0,00118 |
| BF | GO:0000723 | telomere maintenance | 4 | 3 | 0,76 | 0,00118 |
| BF | GO:0009058 | biosynthetic process | 873 | 132 | 165,93 | 0,00244 |
| BF | GO:0006355 | regulation of transcription, DNA-templat... | 297 | 33 | 56,45 | 0,00282 |
| BF | GO:0006364 | rRNA processing | 22 | 9 | 4,18 | 0,0032 |
| BF | GO:0006470 | protein dephosphorylation | 35 | 9 | 6,65 | 0,00323 |
| CC | GO:0008305 | integrin complex | 3 | 3 | 0,6 | 0,0003 |
| CC | GO:0030127 | COPII vesicle coat | 3 | 3 | 0,6 | 0,0003 |
| CC | GO:0005615 | extracellular space | 9 | 4 | 1,81 | 0,002 |
| CC | GO:0005783 | endoplasmic reticulum | 47 | 11 | 9,46 | 0,0022 |
| CC | GO:0030117 | membrane coat | 21 | 9 | 4,23 | 0,0027 |
| CC | GO:0000775 | chromosome, centromeric region | 7 | 3 | 1,41 | 0,0045 |
| CC | GO:0005672 | transcription factor TFIIA complex | 2 | 2 | 0,4 | 0,0045 |
| CC | GO:0072669 | tRNA-splicing ligase complex | 2 | 2 | 0,4 | 0,0045 |
| CC | GO:0005694 | chromosome | 54 | 15 | 10,87 | 0,0051 |
| CC | GO:0042025 | host cell nucleus | 73 | 10 | 14,69 | 0,0236 |
| CC | GO:0000808 | origin recognition complex | 4 | 2 | 0,81 | 0,0248 |
| CC | GO:0030286 | dynein complex | 18 | 4 | 3,62 | 0,0292 |
| CC | GO:0005840 | ribosome | 126 | 15 | 25,36 | 0,0305 |
| MF | GO:0005515 | protein binding | 1576 | 283 | 289,43 | < 1e-30 |
| MF | GO:0005524 | ATP binding | 593 | 127 | 108,91 | < 1e-30 |
| MF | GO:0003723 | RNA binding | 202 | 33 | 37,1 | 0,00012 |
| MF | GO:0035091 | phosphatidylinositol binding | 27 | 8 | 4,96 | 0,0002 |
| MF | GO:0003777 | microtubule motor activity | 35 | 9 | 6,43 | 0,00027 |
| MF | GO:0008289 | lipid binding | 56 | 17 | 10,28 | 0,00084 |
| MF | GO:0051015 | actin filament binding | 13 | 5 | 2,39 | 0,00088 |
| MF | GO:0004252 | serine-type endopeptidase activity | 95 | 15 | 17,45 | 0,00097 |
| MF | GO:0019901 | protein kinase binding | 4 | 3 | 0,73 | 0,00099 |
| MF | GO:0042626 | ATPase-coupled transmembrane transporter... | 31 | 8 | 5,69 | 0,00157 |
| MF | GO:0004721 | phosphoprotein phosphatase activity | 42 | 11 | 7,71 | 0,00232 |
| MF | GO:0004435 | phosphatidylinositol phospholipase C act... | 5 | 3 | 0,92 | 0,00236 |
| MF | GO:0004930 | G protein-coupled receptor activity | 96 | 15 | 17,63 | 0,00332 |
| MF | GO:0004386 | helicase activity | 34 | 10 | 6,24 | 0,00335 |
| MF | GO:0032217 | riboflavin transmembrane transporter act... | 2 | 2 | 0,37 | 0,00408 |

**Supplementary File 1E.** The 15 most significant enriched GO-terms per type in DEGs enriched in middle-aged queens in the gaster tissue. BP = Biological processes, CC = Cellular component, and MF = Molecular functions.

| Type | GO.ID | Term | Annotated | Significant | Expected | Fisher |
| --- | --- | --- | --- | --- | --- | --- |
| BF | GO:0006886 | intracellular protein transport | 86 | 23 | 19,64 | 0,00023 |
| BF | GO:0015986 | ATP synthesis coupled proton transport | 12 | 6 | 2,74 | 0,00025 |
| BF | GO:0016192 | vesicle-mediated transport | 91 | 22 | 20,78 | 0,00036 |
| BF | GO:0009166 | nucleotide catabolic process | 5 | 4 | 1,14 | 0,00065 |
| BF | GO:0007166 | cell surface receptor signaling pathway | 57 | 23 | 13,01 | 0,00082 |
| BF | GO:0006270 | DNA replication initiation | 10 | 5 | 2,28 | 0,00086 |
| BF | GO:0045454 | cell redox homeostasis | 15 | 6 | 3,42 | 0,00107 |
| BF | GO:0006096 | glycolytic process | 11 | 5 | 2,51 | 0,00146 |
| BF | GO:0005975 | carbohydrate metabolic process | 127 | 36 | 29 | 0,00189 |
| BF | GO:0006486 | protein glycosylation | 26 | 8 | 5,94 | 0,00226 |
| BF | GO:0009116 | nucleoside metabolic process | 16 | 8 | 3,65 | 0,00229 |
| BF | GO:0006470 | protein dephosphorylation | 35 | 9 | 7,99 | 0,00243 |
| BF | GO:0005978 | glycogen biosynthetic process | 4 | 3 | 0,91 | 0,00245 |
| BF | GO:0007219 | Notch signaling pathway | 4 | 3 | 0,91 | 0,00245 |
| BF | GO:0042176 | regulation of protein catabolic process | 4 | 3 | 0,91 | 0,00245 |
| CC | GO:0005886 | plasma membrane | 53 | 14 | 10,39 | 0,00012 |
| CC | GO:0016592 | mediator complex | 21 | 8 | 4,11 | 0,00014 |
| CC | GO:0005667 | transcription regulator complex | 30 | 13 | 5,88 | 0,00027 |
| CC | GO:0015935 | small ribosomal subunit | 13 | 6 | 2,55 | 0,00028 |
| CC | GO:0000502 | proteasome complex | 21 | 15 | 4,11 | 0,00185 |
| CC | GO:0000439 | transcription factor TFIIH core complex | 4 | 3 | 0,78 | 0,00197 |
| CC | GO:0015934 | large ribosomal subunit | 13 | 7 | 2,55 | 0,00222 |
| CC | GO:0045261 | proton-transporting ATP synthase complex... | 5 | 3 | 0,98 | 0,00463 |
| CC | GO:0031982 | vesicle | 25 | 10 | 4,9 | 0,00635 |
| CC | GO:0062023 | collagen-containing extracellular matrix | 2 | 2 | 0,39 | 0,00651 |
| CC | GO:0046658 | anchored component of plasma membrane | 2 | 2 | 0,39 | 0,00651 |
| CC | GO:0005853 | eukaryotic translation elongation factor... | 2 | 2 | 0,39 | 0,00651 |
| CC | GO:0032039 | integrator complex | 2 | 2 | 0,39 | 0,00651 |
| CC | GO:0005581 | collagen trimer | 2 | 2 | 0,39 | 0,00651 |
| CC | GO:0016593 | Cdc73/Paf1 complex | 2 | 2 | 0,39 | 0,00651 |
| MF | GO:0005515 | protein binding | 1576 | 475 | 375,85 | < 1e-30 |
| MF | GO:0005524 | ATP binding | 593 | 158 | 141,42 | < 1e-30 |
| MF | GO:0005096 | GTPase activator activity | 53 | 17 | 12,64 | 0,0002 |
| MF | GO:0003924 | GTPase activity | 104 | 21 | 24,8 | 0,00035 |
| MF | GO:0016757 | glycosyltransferase activity | 84 | 24 | 20,03 | 0,0006 |
| MF | GO:0004713 | protein tyrosine kinase activity | 32 | 13 | 7,63 | 0,00061 |
| MF | GO:0050660 | flavin adenine dinucleotide binding | 51 | 14 | 12,16 | 0,00061 |
| MF | GO:0009055 | electron transfer activity | 43 | 15 | 10,25 | 0,00061 |
| MF | GO:0004784 | superoxide dismutase activity | 3 | 3 | 0,72 | 0,00074 |
| MF | GO:0004017 | adenylate kinase activity | 3 | 3 | 0,72 | 0,00074 |
| MF | GO:0004449 | isocitrate dehydrogenase (NAD+) activity | 3 | 3 | 0,72 | 0,00074 |
| MF | GO:0004674 | protein serine/threonine kinase activity | 55 | 18 | 13,12 | 0,00075 |
| MF | GO:0004177 | aminopeptidase activity | 9 | 6 | 2,15 | 0,00185 |
| MF | GO:0005085 | guanyl-nucleotide exchange factor activi... | 33 | 9 | 7,87 | 0,00204 |
| MF | GO:0016491 | oxidoreductase activity | 377 | 88 | 89,91 | 0,00209 |

**Supplementary File 1F. Shared enriched GO-terms for both tissues (head-thorax and gaster) enriched in *prope mortem* queens.**

| Type | GO.ID | Term |
| --- | --- | --- |
| BF | GO:0007186 | G protein-coupled receptor signaling pat... |
| BF | GO:0007264 | small GTPase mediated signal transductio... |
| BF | GO:0007018 | microtubule-based movement |
| BF | GO:0006364 | rRNA processing |
| BF | GO:0016579 | protein deubiquitination |
| BF | GO:0006506 | GPI anchor biosynthetic process |
| BF | GO:0042157 | lipoprotein metabolic process |
| BF | GO:0007178 | transmembrane receptor protein serine/th... |
| BF | GO:0006438 | valyl-tRNA aminoacylation |
| BF | GO:0001522 | pseudouridine synthesis |
| BF | GO:0006303 | double-strand break repair via nonhomolo... |
| BF | GO:0006432 | phenylalanyl-tRNA aminoacylation |
| BF | GO:0006511 | ubiquitin-dependent protein catabolic pr... |
| BF | GO:1901642 | nucleoside transmembrane transport |
| BF | GO:0006265 | DNA topological change |
| BF | GO:0034968 | histone lysine methylation |
| BF | GO:0006406 | mRNA export from nucleus |
| BF | GO:0006397 | mRNA processing |
| CC | GO:0005615 | extracellular space |
| CC | GO:0030117 | membrane coat |
| CC | GO:0030286 | dynein complex |
| MF | GO:0005515 | protein binding |
| MF | GO:0035091 | phosphatidylinositol binding |
| MF | GO:0008289 | lipid binding |
| MF | GO:0051015 | actin filament binding |
| MF | GO:0019901 | protein kinase binding |
| MF | GO:0042626 | ATPase-coupled transmembrane transporter... |
| MF | GO:0004435 | phosphatidylinositol phospholipase C act... |
| MF | GO:0004930 | G protein-coupled receptor activity |
| MF | GO:0004832 | valine-tRNA ligase activity |
| MF | GO:0008017 | microtubule binding |
| MF | GO:0003682 | chromatin binding |
| MF | GO:0004402 | histone acetyltransferase activity |
| MF | GO:0004725 | protein tyrosine phosphatase activity |
| MF | GO:0003755 | peptidyl-prolyl cis-trans isomerase acti... |
| MF | GO:0003700 | DNA-binding transcription factor activit... |
| MF | GO:0016817 | hydrolase activity, acting on acid anhyd... |
| MF | GO:0005337 | nucleoside transmembrane transporter act... |
| MF | GO:0004826 | phenylalanine-tRNA ligase activity |
| MF | GO:0009982 | pseudouridine synthase activity |
| MF | GO:0004970 | ionotropic glutamate receptor activity |
| MF | GO:0002161 | aminoacyl-tRNA editing activity |
| MF | GO:0004675 | transmembrane receptor protein serine/th... |
| MF | GO:0003950 | NAD+ ADP-ribosyltransferase activity |

**Supplementary File 1G. Shared enriched GO-terms for both tissues (head-thorax and gaster) enriched in middle-aged queens.**

| Type | GO.ID | Term |
| --- | --- | --- |
| BF | GO:0045454 | cell redox homeostasis |
| BF | GO:0006096 | glycolytic process |
| BF | GO:0005975 | carbohydrate metabolic process |
| BF | GO:0006470 | protein dephosphorylation |
| BF | GO:0009966 | regulation of signal transduction |
| BF | GO:0006614 | SRP-dependent cotranslational protein ta... |
| BF | GO:0045944 | positive regulation of transcription by ... |
| BF | GO:0016570 | histone modification |
| BF | GO:0071985 | multivesicular body sorting pathway |
| BF | GO:0120009 | intermembrane lipid transfer |
| BF | GO:0042256 | mature ribosome assembly |
| BF | GO:0006368 | transcription elongation from RNA polyme... |
| BF | GO:0043161 | proteasome-mediated ubiquitin-dependent ... |
| BF | GO:0006415 | translational termination |
| BF | GO:0007186 | G protein-coupled receptor signaling pat... |
| BF | GO:0051603 | proteolysis involved in cellular protein... |
| BF | GO:0009435 | NAD biosynthetic process |
| BF | GO:0006879 | cellular iron ion homeostasis |
| BF | GO:0007179 | transforming growth factor beta receptor... |
| BF | GO:0006633 | fatty acid biosynthetic process |
| BF | GO:0006413 | translational initiation |
| BF | GO:0008299 | isoprenoid biosynthetic process |
| CC | GO:0016592 | mediator complex |
| CC | GO:0015934 | large ribosomal subunit |
| CC | GO:0045261 | proton-transporting ATP synthase complex... |
| CC | GO:0062023 | collagen-containing extracellular matrix |
| CC | GO:0046658 | anchored component of plasma membrane |
| CC | GO:0016593 | Cdc73/Paf1 complex |
| CC | GO:0000814 | ESCRT II complex |
| CC | GO:0005839 | proteasome core complex |
| CC | GO:0022625 | cytosolic large ribosomal subunit |
| CC | GO:0048500 | signal recognition particle |
| CC | GO:0005779 | integral component of peroxisomal membra... |
| CC | GO:0005739 | mitochondrion |
| MF | GO:0005515 | protein binding |
| MF | GO:0005524 | ATP binding |
| MF | GO:0003924 | GTPase activity |
| MF | GO:0016757 | glycosyltransferase activity |
| MF | GO:0009055 | electron transfer activity |
| MF | GO:0004784 | superoxide dismutase activity |
| MF | GO:0004017 | adenylate kinase activity |
| MF | GO:0004449 | isocitrate dehydrogenase (NAD+) activity |
| MF | GO:0004177 | aminopeptidase activity |
| MF | GO:0003714 | transcription corepressor activity |
| MF | GO:0003747 | translation release factor activity |
| MF | GO:0004089 | carbonate dehydratase activity |
| MF | GO:0048038 | quinone binding |
| MF | GO:0004719 | protein-L-isoaspartate (D-aspartate) O-m... |
| MF | GO:0120013 | lipid transfer activity |
| MF | GO:0004421 | hydroxymethylglutaryl-CoA synthase activ... |
| MF | GO:0008312 | 7S RNA binding |
| MF | GO:0046933 | proton-transporting ATP synthase activit... |
| MF | GO:0051539 | 4 iron, 4 sulfur cluster binding |
| MF | GO:0030246 | carbohydrate binding |
| MF | GO:0008235 | metalloexopeptidase activity |
| MF | GO:0004930 | G protein-coupled receptor activity |
| MF | GO:0008138 | protein tyrosine/serine/threonine phosph... |
| MF | GO:0016409 | palmitoyltransferase activity |
| MF | GO:0004114 | 3',5'-cyclic-nucleotide phosphodiesteras... |
| MF | GO:0008199 | ferric iron binding |

**Supplementary File 1H. Deviance Information Criterion of three tested models (logistic, Gompertz and Weibull) for the age-specific mortality of ant queens.** Using the function multibasta of the R package BaSTA (Survival Bayesian Trajectory Analysis, v. 1.9.5). The model with the lowest DIC value is assumed to provide the best fit.

| Model | Shape | DIC average | DIC mode | pD | k | DIC | Difference in DIC | Rank |
| --- | --- | --- | --- | --- | --- | --- | --- | --- |
| Gompertz | simple | 1596 | 1454 | 71.0 | 3 | 1667 | 0 | 1 |
| Logistic | simple | 1592 | 1438 | 77.1 | 4 | 1669 | 2.26 | 2 |
| Weibull | simple | 1616 | -20798 | 11207.1 | 3 | 12823 | 11156.68 | 3 |

**Supplementary File 1I. Estimated coefficients of age-specific mortality of ant queens.** Using a logistic model and the R package BaSTA (Survival Bayesian Trajectory Analysis, v. 1.9.5).

| Parameter | Estimate | Standard Error | Lower 95% confidence interval | Upper 95% confidence interval | Se Auto cor | Update Rate | Pot Scale Reduc |
| --- | --- | --- | --- | --- | --- | --- | --- |
| b_0_ | -7.37 | 0.23 | -7.85 | -6.96 | 0.61 | 0.25 | 1 |
| b_1_ | 0.02 | 1.1*10^-3^ | 0.01 | 0.02 | 0.60 | 0.25 | 1 |
| pi.1 | 0.15 | 2.2*10^-3^ | 0.14 | 0.15 | 0.01 | 1 | 1 |
